# Estimating plant–insect interactions under climate change with limited data

**DOI:** 10.1038/s41598-022-14625-9

**Published:** 2022-07-06

**Authors:** Yui Tamura, Takeshi Osawa, Ken Tabuchi, Kazuhisa Yamasaki, Tokumitsu Niiyama, Shigeto Sudo, Yasushi Ishigooka, Akira Yoshioka, Mayura B. Takada

**Affiliations:** 1 Graduate School of Urban Environmental Sciences, Tokyo Metropolitan University, Minami-Osawa 1-1, Hachiouji, Tokyo 192-0397 Japan; 2 Division of Agro-Environment Research, Tohoku Agricultural Research Center, NARO, Morioka, Japan; 3 Faculty of Agriculture, Tokyo University of Agriculture and Technology, Fuchu, Tokyo Japan; 4 Akita Plant Protection Office, Akita, Japan; 5 Institute for Agro-Environmental Sciences, National Agriculture and Food Research Organization, NARO, Tsukuba, Japan; 6 Fukushima Regional Collaborative Research Center, National Institute for Environmental Studies, Miharu, Japan; 7 Faculty of Science and Engineering, Chuo University, Tokyo, Japan

**Keywords:** Agroecology, Climate-change ecology

## Abstract

Climate change may disrupt species–species interactions via phenological changes in one or both species. To predict and evaluate the influence of climate change on these interactions, long-term monitoring and sampling over large spatial areas are required; however, funding and labor constraints limit data collection. In this study, we predict and evaluate the plant–insect interactions with limited data sets. We examined plant–insect interaction using observational data for development of the crop plant rice (*Oryza sativa*) and an effective accumulated temperature (EAT) model of two mirid bugs (*Stenotus rubrovittatus* and *Trigonotylus caelestialium*). We combined 11 years of records monitoring rice phenology and the predicted phenology of mirid bugs using spatially–explicit EAT models based on both spatially and temporally high resolutions temperature data sets, then evaluated their accuracy using actual pest damage records. Our results showed that the predicted interactions between rice and mirid bugs explained rice damage to some degree. Our approach may apply predicting changes to plant–insect interactions under climate change. As such, combining plant monitoring records and theoretical predictions of insect phenology may be effective for predicting species–species interactions when available data are limited.

## Introduction

The Intergovernmental Panel on Climate Change report mentions that many natural systems are affected by regional climate changes, particularly temperature increases^1^. Many ecological studies have demonstrated that spring and summer phenological events now occur earlier in the year than previously^2^, and responses in terms of phenological change of biological systems have been well documented^3–6^. For example, plant species change the timing of their blooming and fruit set^5,7,8^, insect species change their life cycle such as the timing of hatching and maturation^9,10^, and bird species change the timing of their egg hatching and migration periods^9,11,12^.

Responses by individual species to climate change may disrupt their interactions with other species, such as competition, symbioses, and trophic cascades^3,13^. Some studies have shown that recent climate change has caused phenological mismatches between plants and pollinators^14,15^, and mismatches between emerging food resources and herbivorous animals^16,17^, which could directly affect species fitness. Additionally, phenology sensitivities to climate change vary across taxa and trophic levels^2,18^; thus, there are likely still many undetected and/or potential phenological changes across species interactions^13^.

To identify the effects of climate change on species interactions, researchers should first detect the existing species interaction, then evaluate the effects of climate change in each species, and the combined changes of these different ecological processes^19^. However, even when considering the effects of climate change on a single ecological process, there is a need to use long–term studies to evaluate individual fitness^2,19,20^. Moreover, strong inferences on the impacts of climate change require data that covers both long time spans and large spatial scales^13,21,22^; however, funding and labor constraints often limit the extent of data collection^21^.

The phenology of poikilotherms, especially invertebrates like insects, is directly and strongly affected by climate factors, especially temperature^23^. As such, the lifecycle phenology of invertebrate species could be predicted using time-series temperature data sets with their effective accumulated temperature (EAT) model^24,25^. The idea of EAT model encompasses that temperature responses of a particular species, in which a specific amount of thermal units should accumulated above a temperature threshold are required to complete a certain developmental event^23^. In fact, many studies have predicted the lifecycle phenology of insects using the EAT model^24,26–29^. Using this approach, we are able to predict the lifecycle phenology of insects over long time spans and large spatial scales at the landscape level, without detailed monitoring records, using only temperature data that has been compiled at a high spatial and temporal resolution^24^.

The influence of climate change on agricultural production is a central theme in terms of the adaptation to climate change^30,31^. Changes in the interactions between crop plants and pest insects, because of climate change, are an important factor that directly influences crop production. In an agricultural system, the phenology of crop plants such as plant timings and flowerings are often recorded by farmers and/or advisory organizations, and studies of agricultural pest insects have led to established parameters of the EAT model for pesticide management^32^. Thus, the crop plant-pest insect interaction is an ideal system in which to test the changing species interactions caused by climate change using a limited data set, namely using both cropping records and temperature data.

In this study, we predict and evaluate the changing plant–insect interactions using a combination of observation data from crop plants and the EAT model for pest insects. As a case study, we focused on the interaction between rice (*Oryza sativa*) and two mirid insects (*Stenotus rubrovittatus* and *Trigonotylus caelestialium*); both are grain-feeding pests that damage rice in East Asia, as well as other economically-important grains in Japan^33^. Rice is one of the major crops in monsoon Asia. In Japan, there are standard cropping schedules because of clear seasonality: rice cropping occurs once per year, and rice phenology is often recorded (i.e., growth situation in each stage such as ear emergence and maturity). Mirid bugs are hemimetabolous and live on the plant body of open fields i.e., not tunnels under the plant body; thus, their phenology is likely to be directly affected by environmental temperatures. In fact, EAT models for these species have already been established in some regions and have been reported to have good performance^34,35^. Moreover, previous studies suggest that these species are sensitivity to climate change^24,36^. Further, previous studies suggested that these species have relatively low preferences and growth performances for rice^33,37^, thus, phenological matching for rice could be an essential factor for rice damage. As such, first, we evaluated the phenological change in rice using 11 years of monitoring records. Then, we predicted the phenological change in mirid bugs using EAT models. Finally, we predicted the change in the interaction i.e., phenological matching between rice and mirid bugs, combined these interactions, and evaluated their accuracy using actual pest damage records.

## Results

### Change in rice phenology

**Figure 1:**
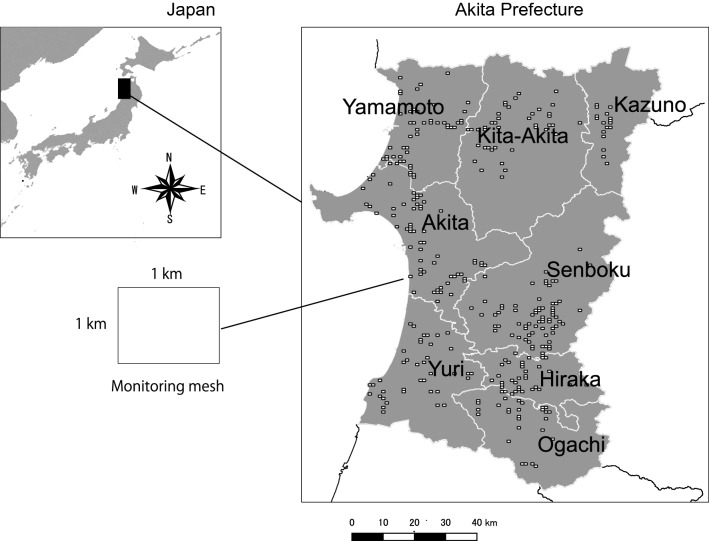
Location and analytical unit used in this study.

The number of monitoring points for the phenology of rice in each region is shown in Table 1. In each region in each year, the number of monitoring paddy fields have variety, specifically their ranged from 4 (minimum) to 24 (maximum), but the distribution of these could cover the area in each region (Fig. 1). Some areas that have no monitoring points do not have paddy fields, dominated forest, or urbanized area.

**Table 1:** Monitoring points for rice phenology in each region in each year.

Region	2003	2004	2005	2006	2007	2008	2009	2010	2011	2012	2013
Akita	21	21	22	22	22	22	18	18	18	18	18
Hiraka	14	14	14	14	14	14	12	11	11	11	11
Kazuno	8	8	7	7	7	7	6	4	4	4	4
Kita-Akita	13	13	13	13	13	13	11	12	12	12	12
Ogachi	11	11	11	11	11	11	9	7	7	7	7
Senboku	24	24	24	24	24	24	20	21	21	21	21
Yamamoto	15	15	15	15	15	15	12	15	15	15	15
Yuri	14	14	14	14	14	14	12	12	12	12	12
Total	120	120	120	120	120	120	100	100	100	100	100

**Figure 2:**
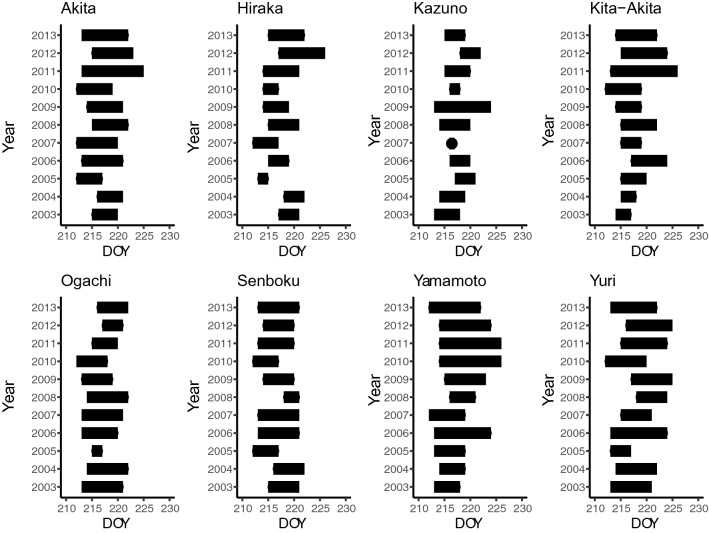
The term of the rice ear emergence date according to the region and year. X axis indicates DOY means “Day of Year” which is continuous number from 1 (1, Jan) to 365 or 366 (31, Dec). The length of each bar indicates the range from the earliest to the latest ear emergence dates in each region in each year.

Figure 2 shows the date of rice ear emergence in each region in each year, wherein the length of each bar indicates the period from the earliest to the latest date. There were no clear trends in terms of earlier or late occurrence over time (Fig. 2). Within the region, the length of ear emergence timing varied greatly (Fig. 2). Generalized linear mixed effects model (GLMM) analysis showed that the median of ear emergence dates did not correlate with the year during the study term, namely higher AIC values compared with the null model, and *p* value of coefficients was relatively high (0.31) (Table 2).

**Table 2:** Generalized linear mixed effects model (GLMM) for the ear emergence date of rice, attacking terms of two bugs over the whole study term (2003–2013). Objective variables were median of that event dates, explanatory variable was the year as continuous value.

Objective variables	Coefficient	Intercept	AIC	*p* value
	(S.E.)	(S.E.)	(null model AIC)	
Ear emergence date of rice	0.07	− 3.12	362.99	0.31
	(0.07)	(1.05)	(357.87)	
Adult term of *S. rubrovittatus*	− 0.37	972.54	449.41	6.97 × 10^–5^
	(0.09)	(177.41)	(460.51)	
Nymphal term of *T. caelestialium*	− 0.597	1433.61	495.69	1.87 × 10^–6^
	(0.12)	(232.87)	(514.36)	

*p* value indicates the result for t-statistics using Satterthwaite's method for denominator degrees of freedom.

### Change of the estimated phenology of mirid bugs

**Figure 3:**
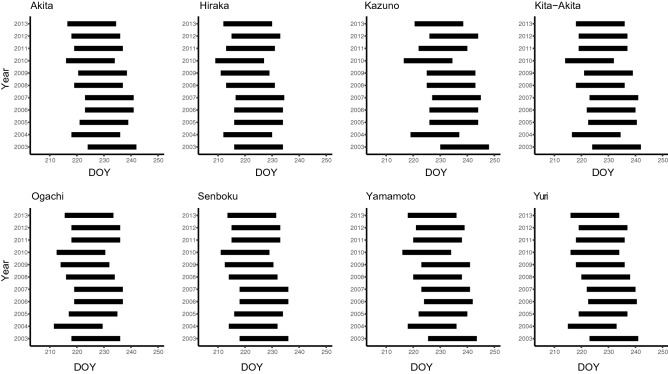
The theoretical length of time from the adult stage of the second generation of *S. rubrovittatus* for each year in each region. X axis indicates DOY means “Day of Year” which is continuous number from 1 (1, Jan) to 365 or 366 (31, Dec). The length of each bar indicates 18 days, the mean longevity of this species.

**Figure 4:**
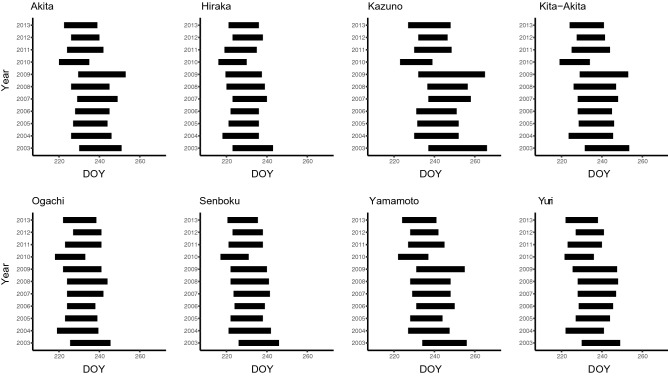
The theoretical length from the nymphal stage of the third generation of *T. caelestialium* for each year in each region. X axis indicates DOY means “Day of Year” which is continuous number from 1 (1, Jan) to 365 or 366 (31, Dec). The length of each bar indicates the period from the median egg hatching date across regions to the median maturation date across the region in the year.

Figure 3 shows the predicted phenology of *S. rubrovittatus*, with a specific length of 18 days from the median of adult emergence date in the second generation in each year in each region. Figure 4 shows the length of the juvenile stage of the third generation of *T. caelestialium*; the length of each bar indicates the range covering the median egg hatching date within the region to the median maturation date within the region in a given year. In short, the predicted phenological trends of mirid bugs were synchronized across regions (Figs. 3, 4). For example, the phenology of *S. rubrovittatus* was relatively faster in both 2004 and 2010 for all regions (Fig. 3), whereas that of *T. caelestialium* was relatively faster in 2010 and relatively slower in 2003 for all regions (Fig. 4). Actually, GLMM analysis showed that both the median of adult emergence date of the second generation on *S. rubrovittatus* and juvenile stage of the third generation on *T. caelestialium* were negatively correlated with the year during the study term, namely lower AIC values compared with the null model, and *p* value of coefficients was too low (p < 0.001) (Table 2). Thus, the phenology of the species tended to become earlier over time.

### Change in the interaction between rice and mirid bugs

**Figure 5:**
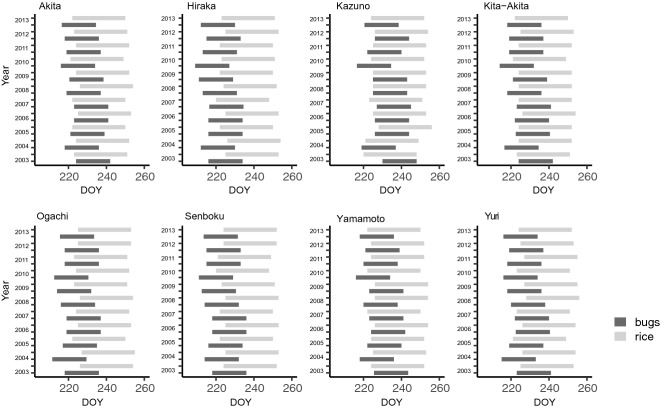
The overlap between the vulnerable term of rice (16 days in total, from 7 to 23 days after the median date of ear emergence) and the attacking term of *S. rubrovittatus* (the adult stage in the second generation) for each year in each region. X axis indicates DOY means “Day of Year” which is continuous number from 1 (1, Jan) to 365 or 366 (31, Dec).

**Figure 6:**
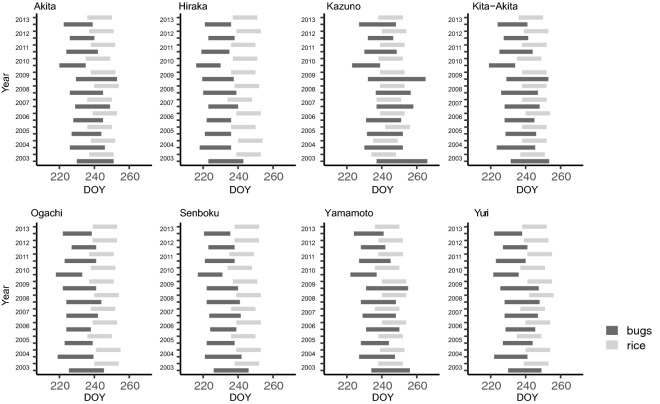
The overlap between the vulnerable term of rice (14 days in total, from 21 to 35 days from the ear emergence date) and the attacking term of *T. caelestialium* (the nymphal stage) for each year in each region. X axis indicates DOY means “Day of Year” which is continuous number from 1 (1, Jan) to 365 or 366 (31, Dec).

Figures 5 and 6 show the overlapping trends between the vulnerable term of rice and the predicted attacking term of each species. For *S. rubrovittatus*, there was substantial overlap between the vulnerable term for rice and the predicted attacking term of the bugs (Fig. 5), whereas for *T. caelestialium*, there were relatively few overlapping days (Fig. 6). Across the eight regions, almost all the attacking terms of *S. rubrovittatus* in Akita, Kazuho, Kita-Akita, and Yamamoto overlapped with the vulnerable term for rice (Fig. 5). Conversely, for *T. caelestialium*, there was a relatively large number of overlapping days in Kazuho, compared with the other regions, and both Ogachi and Senboku had few overlapping days (Fig. 6). For both *S. rubrovittatus* (Fig. 5) and *T. caelestialium* (Fig. 6), the number of overlapping days varied across regions and years.

The GLMM for the occurrence of rice damage revealed that the overlapping date of both bugs were positively correlated with the occurrence of rice damage but AIC values were higher than that of the null model (Table 3). Moreover, the statistical test revealed that the *p* values of overlapping dates for both species were relatively high, 0.31 and 0.33, respectively (Table 3). For the divided periods from 2003 to 2005, the *T. caelestialium* overlapping date was positively correlated with the occurrence of rice damage with approximately 3 lower AIC compared with that of null model (Table 4). Further, the statistical test of that model showed that *p* values of overlapping date was low (0.08) (Table 4). In contrast, the *S. rubrovittatus* overlapping date was negatively related with the occurrence of rice damage, with approximately 2 higher AIC value compared with that of the null model, and high *p* values (0.92) on statistical tests (Table 4).

**Table 3:** Generalized linear mixed effects model (GLMM) for the occurrence of crop damage over the whole study term (2003–2013). Objective variables were occurrence of crop damage, explanatory variable were overlapping days.

Explanatory variables	Coefficient	Intercept	AIC	*p* value
	(S.E.)	(S.E.)		
Overlapping of adult term of *S. rubrovittatus* with ear emergence of rice	0.07	− 3.12	69.32	0.31
	(0.07)	(1.05)		
Overlapping of nymphal term of *T. caelestialium* with ear emergence of rice	0.08	− 2.75	69.47	0.33
	(0.08)	(0.798)		
Null model	–	− 2.27	68.47	
		(0.55)		

*p* value indicates the result for t-statistics using Satterthwaite's method for denominator degrees of freedom.

**Table 4:** Generalized linear mixed effect model (GLMM) for the occurrence of crop damage over the two divided study periods (2003–2005 and 2006–2013). Objective variables were occurrence of crop damage, explanatory variable were overlapping days.

Monitoring term	Explanatory variables	Coefficient	Intercept	AIC	*p* value
		(S.E.)	(S.E.)	(delta AIC with null model)	
2003 – 2005 (*T. caelestialium* dominant)	Overlapping of adult term of *S. rubrovittatus* with ear emergence of rice	− 0.92 × 10^–2^	− 1.84	24.08	0.92
		(0.95 × 10^–1^)	(1.25)	(1.99)	
	Overlapping of nymphal term of *T. caelestialium* with ear emergence of rice	0.32	− 4.73	**19.07**	0.08
		(0.18)	(2.11)	**(− 3.02)**	
	Null model	–	− 1.95	22.09	
			(0.62)		
2006 – 2013 (*S. rubrovittatus* dominant)	Overlapping of adult term of *S. rubrovittatus* with ear emergence of rice	0.16	− 4.55	**48.89**	0.16
		(0.12)	(1.85)	**(− 0.69)**	
	Overlapping of nymphal term of *T. caelestialium* with ear emergence of rice	− 0.03	− 2.33	51.50	0.79
		(0.10)	(0.91)	(1.92)	
	Null model	–	− 2.27	49.58	
			(0.55)		

Bold indicated the lower AIC value compare with that of null model. *p* value indicates the result for t-statistics using Satterthwaite's method for denominator degrees of freedom.

Conversely from 2006 to 2013, the overlapping date for *S. rubrovittatus* was positively correlated for the occurrence of rice damage, with approximately 0.7 lower AIC compared with that of the null model, low *p* values (0.16) on statistical tests (Table 4). In contrast, the *T. caelestialium* overlapping date was negatively related with the occurrence of rice damage, with approximately 2 higher AIC value compared with that of the null model, and high *p* values (0.79) on statistical tests (Table 3).

## Discussion

This study predicted the plant–insect interactions using a combination of observational data on crop plant rice and the estimated phenology of pest insects (*S. rubrovittatus* and *T. caelestialium*) using an EAT model. We then evaluated the accuracy of these predictions using pest damage records. The predicted interactions between rice and mirid bugs explained rice damage, to some degree. Our approach may prove successful in predicting plant–insect interactions with limited data.

### Phenological change of rice

We used monitoring records of rice phenology over the 11 years from 2003 to 2013; these covered almost all of Akita Prefecture. Over this term, we could not find any clear trends over time. Previous studies have suggested that the phenology of plants such as flowering and fruit set could shift earlier with climate change^5,7,8^. However, these studies were conducted over long time spans of more than three decades on a large spatial scale (e.g., continental scale). To directly detect changes to the phenology of plants, our data may be inadequate, both in terms of the monitoring term and spatial extent^21,22^. Moreover, the phenology of crop plants could change according to agricultural activities such as planting time and the seedling raising term. These factors may obscure phenological changes, even under climate change.

### Phenological change in mirid bugs

We predicted the theoretical phenology of two mirid bug species over 11 years, predicting that the phenology of these species are likely to occur earlier over this time span. In general, insect species are highly sensitive to climate change because they are poikilothermic and have a small body size^38^. Moreover, several studies have shown phenological changes of them with climate change^38–41^. Importantly, our results suggested that phenological change of insects could occur even 11 years later, which is a short duration compared with previous studies^38–41^. One possible explanation on that is relating their ecological characteristic, such as hemimetabolous. Studies on phenological changes often used holometabolous species, such as butterflies, because of their detectability^39–41^. Although we used theoretical prediction values, our results suggest that phenological changes, such as an earlier lifecycle, can occur even over a relatively-short term of 11 years for hemimetabolous insects.

### Phenological changes to the interaction between rice and mirid bugs

The number of overlapping days between the vulnerable term of rice to bugs and the attacking term of bugs varied across the mirid bug species and years. For the 11-year analysis, the overlapping date number for two bugs were positively correlated with the occurrence of rice damage. For the analysis of divided terms, the overlapping date number for *T. caelestialium* from 2003 to 2005 and that of *S. rubrovittatus* from 2006 to 2013 were positively correlated with the occurrence of rice damage. Thus, our approach to predicting plant–insect interactions using a combination of observational and theoretical data, may to some degree reflect real world situations. Notably, the GLMM models in which the study term was divided into two periods showed good performance. More specifically, the *T. caelestialium* model showed good performance for 2003–2005, when *T. caelestialium* was the dominant species^42^, whereas the *S. rubrovittatus* model showed good performance from 2006 to 2013, when *S. rubrovittatus* was the dominant species^42^. For *S. rubrovittatus*, the overlapping date number in each year did not change across study years, whereas for *T. caelestialium*, it tended to reduce the overlapping date number the later term, especially after 2010. Although the dominant bug species changed from *T. caelestialium* to *S. rubrovittatus* in Akita Prefecture^42^, the interaction between *T. caelestialium* and rice, for example the chance of bug attacks might reduce over the same term due to climate factors. Conversely, the attack by *S. rubrovittatus* for rice remained stable; thus, it may cause consistent damage to rice, compared to *T. caelestialium*.

## Conclusion

To predict plant–insect interactions with limited data, our approach, combining monitoring records of plants and theoretical predictions of insect phenology, proved to be effective. This approach could be applied over a relatively fine scale, compared with previous studies^13,21,22^. Additionally, our approach can also be applied to situations with real insect records and theoretical predictions of plant phenology. For theoretical predictions, we can also use parameters other than temperature, such as water condition, to predict the phenology (e.g.^43^) and future these (e.g.^44^). Our approach could contribute to predicting complex species interactions under climate change.

## Methods

### Study area

This study was conducted in Akita Prefecture, Japan (39° 43′ N, 140° 6′ E, 11,637.52 km^2^; Fig. 1), with a mean annual precipitation of 1,741.6 mm, including heavy snow, and a mean annual temperature of 12.1 °C ^66^. The study area was in the Tohoku region of Japan, on the Sea of Japan side of the country (Fig. 1). It is dominated by rice-producing farms who practice in paddy fields with small crop varieties, namely dominated by one variety: “Akitakomachi”^65^.

Akita Prefecture is divided into eight regions, each with their own municipality (Fig. 1). We used each region as an analytical unit since it reflects the location conditions (e.g., seaside, mountainous zone) that may influence the phenology of both rice and mirid bugs.

We used a grid size of approximately 1 km^2^, hereafter referred to as 1-km mesh, as the basic unit (Fig. 1). The 1-km mesh system is a standard Japanese unit used for several types of statistics^67^. The locations of the 1-km mesh were determined arbitrarily by the Japanese government for comparing statistics such as populations and age structures.

### Growth phenology of rice

The growth phenology records of rice were derived from the monitoring program of the Akita Plant Protection Office. In this program, an individual observed the growth stages of rice in selected paddy fields approximately two times per month during the rice growing season from 2003 to 2013; this encompassed 120 monitoring paddy fields from 2003 to 2008 and 100 monitoring paddy fields from 2009 to 2013 (Table 1). Monitoring paddy fields were not fixed for the observation term and could be changed each year; thus, we merged these records from each of the eight regions to treat the monitoring paddy fields as sampling points within each region. Based on the monitoring data, we extracted records of ear emergence dates since mirid bugs aggressively attack rice after ear emergence^45–47^. The 1-km mesh units that included at least one monitoring paddy field are shown in Fig. 1. To test the overall trend of ear emergence dates during the study term, we used a generalized linear mixed-effect model (GLMM) with a Gaussian distribution; the median of ear emergence dates for each year in each region was the objective variable, whereas the year as a continuous value was the explanatory variable. We used the region as a random effect term. If the coefficient of an explanatory variable was negative, it suggests that the timing of ear emergence could be earlier.

### Pest insect species

As a case study, we used the mirid bugs *S. rubrovittatus* and *T. caelestialium*. In Akita Prefecture, both species were common, but the dominant species changed from *T. caelestialium* to *S. rubrovittatus* during the study term^42^. Worldwide, grain-feeding bugs cause major damage to rice (*O. sativa*) and other economically-important grains^33^. In Japan, the discoloration of rice grains caused by mirid bugs is a serious economic problem for rice cultivation^25,48–50^ because the contamination of damaged rice with discolored grains results in a lower grade under Japanese rice quality regulations and thus a lower market price. Since the 1990s, mirid bugs, including both *S. rubrovittatus* and *T. caelestialium*, have been recognized as major rice pests in the Tohoku region, including Akita Prefecture, and have caused economic damage to rice farmers^42,51^.

### Daily temperature data in each 1-km mesh

We used the maximum and minimum daily temperature for each 1-km mesh (hereafter NIAES mesh) to calculate the EAT. This data set was created from daily observed meteorological data obtained at the Automated Meteorological Data Acquisition System stations and the published data set Mesh Climatic Data (Japan Meteorological Agency). For detailed descriptions of the method used to calculate the meteorological values of grid cells, see Seino^52^ and Ishigooka et al.^53^.

### Development parameters of both mirid bugs

The temperature parameters for both *S. rubrovittatus* and *T. caelestialium* living in Akita Prefecture were derived from laboratory rearing studies^54^. The developmental zero point and cumulative temperature for *S. rubrovittatus* was 12.42 and 101.98 for egg hatching, 10.73 and 226.297 for the nymphal stage, and 13.17 and 81.80 for sexual maturation, respectively. The developmental zero point and cumulative temperature for *T. caelestialium* was 12.01 and 95.93 for egg hatching, 11.87 and 190.47 for the nymphal stage, and 12.67 and 47.04 for sexual maturation, respectively. All parameters are shown in Appendix Table 1. Using NIAES mesh daily temperature data and these theoretical models, we predicted the date of the development stage, as well as the duration of each stage.

### Predicting the phenology of mirid bugs

Using the NIAES mesh daily temperature data and the development parameters of both *S. rubrovittatus* and *T. caelestialium*, we predicted the life cycle of these species by calculating the EAT from 2003 to 2013 within each 1-km mesh that had at least one monitoring paddy field (Fig. 1). The EAT was calculated using the triangle method^55^, with April 1 as the start date. In this study system, setting the initial date is not sensitive to EAT because the average temperature in April within this region is significantly lower (less than 10 °C) than the developmental zero points of the mirid bugs^66^. First, we predicted the theoretical egg hatching date from April 1 using the EAT of egg hatching of the over-wintering generation. Then, we predicted the duration of the nymphal stage from the date when eggs hatched using the EAT of the nymphal stage. Finally, we predicted the sexual maturation date from the date when the nymphal stage ended using the EAT of sexual maturation. The first and subsequent generation cycles were predicted using the same procedure from the date of sexual maturation (i.e., the start of the oviposition period) of the previous generation. We ignored the shift to the production of diapausing eggs induced by short-day conditions^56^.

For *S. rubrovittatus*, the bug aggressively attacks rice during the adult stage^57^; thus, we used the attacking term of this bug as from the second generation of adult emergence to 18 days; male and female longevity is 13.0 ± 2.9 and 22.2 ± 2.7 (mean ± S.E.) days, respectively, when they are reared on wheat seedlings in a group (n = 12 for each sex; Ogata et al.^58^). For *T. caelestialium*, the bug aggressively attacks rice during the nymphal stage^59^, thus, we used the attacking term of this bug as from the nymphal stage of the third generation, according to a previous study^34^. Therefore, we illustrated the change in these terms predicted by the EAT model from 2003 to 2013 as the phenological change of the bugs in this study. In this study, we used the median of the date of each stage in each region as the starting day of the attacking term because each region had at least one monitoring paddy field.

To test the overall trend of attacking terms of bugs during the study term, we used a GLMM with Gaussian distribution; the median of attacking terms of bugs for each year in each region was the objective variable, whereas the year as the continuous value was the explanatory variable. We used the region as a random effect term. If the coefficient of the explanatory variable was negative, it suggests that the phenology of bugs could be earlier.

### Predicting the interaction between rice and mirid bugs

To predict changes to the interaction between rice and mirid bugs, we calculated the overlapping number of days between the vulnerable term of rice against bugs and the attacking terms of each species, in each region in each year. For the vulnerable term of rice, we used 28 days after the 7 days from ear emergence date for *S. rubrovittatus* because previous studies have shown that *S. rubrovittatus* aggressively attack rice over that term^45,47,60^. Moreover, we used 14 days after the 21 days from the ear emergence date for *T. caelestialium*, based on the results of previous studies^61,62^. We calculated the number of overlapping days, which we used as the magnitude of the interaction between rice and mirid bugs. In this study, we used the median of the date of ear emergence in each region as the starting day of the vulnerable term because each region had monitoring paddy fields.

### Evaluating the accuracy of the estimated species interaction

To evaluate the accuracy of the predicted interaction value, we used the published record of rice damage for each region, which included the ratio of discolored rice grains^42^. Using this record, we established the occurrence of rice damage according to Japanese rice quality regulations^67^; if the ratio of discolored rice grains was greater than 0.1, we considered damage to be present. To evaluate the relationship between the occurrence of rice damage and the index of species interaction (overlapping date), we used a GLMM with a binominal distribution; the occurrence of rice damage for each year in each region was the objective variable, whereas the overlapping date for both species was the explanatory variable. Moreover, we divided the study term into two periods: (1) from 2003 to 2005, when *T. caelestialium* was the dominant species; and (2) from 2006 to 2013, when *S. rubrovittatus* was the dominant species^42^. We analyzed each period using the same GLMM model. We used year as a random effect term; we could not use region as a random effect for the data from 2003 to 2005 since it encompassed 3 years, less than the number of regions. To evaluate the model’s performance based on information theory^63^, we established a null model and compared the value of the Akaike’s Information Criterion. Further, we conducted *t-statistics* test^64^ to test the significance of the explanatory variables. All statistical analyses were preformed using R version 4.1.3 with package “lme4” and “lmerTest”^64^.

## Supplementary Information

Supplementary Information.

## Data Availability

Original data are available from internet (in the text) and contact for corresponding author with reasonable requests.
