# Supplementary material for: Estimating plant–insect interactions under climate change with limited data
Source: Sci Rep. 2022 Jul 6;12:10554. doi: 10.1038/s41598-022-14625-9 (PMC9259679; doi:10.1038/s41598-022-14625-9)
Supplement: Supplementary file 1 — Supplementary Information. [file 41598_2022_14625_MOESM1_ESM.docx]

**Appendix Table1.** Development parameters on 2 mirid bugs.

|  | *Stenotus rubrovittatus* |  |  | *Trigonotylus caelestialium* |  |
| --- | --- | --- | --- | --- | --- |
| Stage | Developmental zero | Cumulative temperature |  | Developmental zero | Cumulative temperature |
| Egg hatching | 12.42 | 101.98 |  | 12.01 | 95.93 |
| Nymphal stage | 10.73 | 226.297 |  | 11.87 | 190.47 |
| Sexual maturing | 13.17 | 81.8 |  | 12.67 | 47.04 |

Development parameters were derived from Yamasaki et al. (2021).
